# Remote assessment of physical function in older people: feasibility, safety and agreement with in-person administration

**DOI:** 10.1093/ageing/afaf266

**Published:** 2025-09-27

**Authors:** Kimberley S van Schooten, Daniel Steffens, Adam Engeler, Meg Letton, Alicia Brown, Amy Perram, Lillian Miles, Michelle Ngo, Kim Delbaere

**Affiliations:** Neuroscience Research Australia, Randwick, New South Wales, Australia; University of New South Wales - Sydney, New South Wales, Australia; Royal Prince Alfred Hospital - Surgical Outcomes Research Centre, Camperdown, New South Wales, Australia; The University of Sydney Central Clinical School - Faculty of Medicine and Health, Sydney, New South Wales, Australia; Neuroscience Research Australia, Randwick, New South Wales, Australia; Neuroscience Research Australia, Randwick, New South Wales, Australia; Neuroscience Research Australia, Randwick, New South Wales, Australia; Neuroscience Research Australia, Randwick, New South Wales, Australia; Neuroscience Research Australia, Randwick, New South Wales, Australia; Neuroscience Research Australia, Randwick, New South Wales, Australia; Neuroscience Research Australia, Randwick, New South Wales, Australia; University of New South Wales - Sydney, New South Wales, Australia

**Keywords:** older adults, telehealth, mobility, remote monitoring, digital health, older people

## Abstract

**Background:**

Physical function is a key health indicator in older people. Interchangeable remote and in-person assessments could improve monitoring flexibility.

**Objective:**

To evaluate feasibility, safety and agreement between remote home and in-person laboratory physical function assessments in community-living older people.

**Methods:**

Thirty-seven people aged 60+ completed remote and in-person assessments of the five-times Sit-to-Stand (5STS), Timed Up-and-Go (TUG), standing balance test, 4 m Walk and Short Physical Performance Battery (SPPB) in counter-balanced order, two days apart. Feasibility was assessed as technological and environmental barriers, safety as adverse events, and agreement using intraclass correlation coefficients (ICCs) and smallest detectable differences (SDD), and based on kappa using clinical cutpoints (κ).

**Results:**

All participants completed the remote assessments without adverse events; 8% experienced minor connectivity issues. In-person performance was better for 5STS, TUG, 4 m Walk and standing balance; SPPB scores were comparable. Agreement was good for 5STS, TUG and standing balance (ICCs: 0.89 [95% confidence interval: 0.79–0.94], 0.85 [0.54–0.94], 0.77 [0.59–0.88], respectively)) and moderate for 4 m Walk and SPPB (ICCs: 0.64 [0.19–0.84] and 0.68 [0.46–0.82], respectively). SDD values for 5STS, TUG and SPPB fell within clinically acceptable ranges; categorical agreement was substantial (κ: 0.65, 0.77 and 0.65, respectively).

**Conclusions:**

Remote 5STS and TUG assessments showed good agreement, supporting their use with existing cut-points. Differences between settings in standing balance and walking speed (affecting SPPB) suggest caution in interpretation. Telehealth offers a feasible, safe option for monitoring physical function, though protocol refinements are needed for walking speed and standing balance.

## Key Points

- Remote assessment of physical function is growing, but evidence of its accuracy compared to in-person testing remains limited.
- This study found that remote assessments of physical function in older people are safe and feasible.
- 5STS and TUG tests showed good agreement between remote and in-person assessments (ICCs: 0.89, 0.85).
- The standing balance test showed good agreement (ICC: 0.77) but limited sensitivity across settings.
- Variation in walking speeds resulted in moderate agreement for the 4 m walk and SPPB (ICCs: 0.64, 0.68), necessitating caution.

## Introduction

Physical function is a critical determinant of health and independence in older people. Declines in physical function are associated with increased risk of falls, disability, hospitalisation and mortality [1–3], making it an important metric for clinical decision-making and research. Widely used physical function assessments, Short Physical Performance Battery (SPPB), five times sit-to-stand (5STS), standing balance, 4-meter walking speed (4 m Walk) and the timed Up-and-Go (TUG) test [4], have been rigorously validated in controlled settings. These tools are used with cut-points for diagnosing conditions such as fall risk [5], sarcopenia [6] or frailty [7], and as continuous measures for monitoring recovery or tracking progress over time [8].

The COVID-19 pandemic accelerated the adoption of telehealth, allowing care and research to continue despite strict safety protocols [9]. However, this shift occurred without fully validating whether remote home assessments could reliably replicate gold-standard in-person laboratory assessments. While telehealth offers flexibility and accessibility, it is essential to confirm that remote assessments produce comparable results to in-person assessments to ensure accuracy in diagnosis and monitoring. Emerging evidence suggests disparities between remote and in-person physical function assessments in older people. A recent systematic review [10] reported poor agreement for SPPB scores, inconsistent findings for walking speed and 5STS and strong agreement for the TUG, highlighting the need for further evaluation. Using remote home and in-person laboratory assessments interchangeably could enhance the flexibility of physical function monitoring, enabling test modalities to be tailored to patient needs and logistical constraints. However, systematic differences between settings could compromise conclusions about functional trajectories or intervention outcomes. This study aimed to evaluate the feasibility, safety and agreement between remote and in-person assessments of key physical function measures in community-living older people.

## Methods

### Participants

Participants (52 expressed interest, 37 were eligible) were recruited from the Neuroscience Research Australia Volunteer Register. Inclusion criteria were aged 60 years and older, living in the community in metropolitan Sydney and access to a device (e.g. mobile phone, tablet, laptop and desktop) with internet connectivity, a camera and audio capabilities. People were excluded if they had severe vision and/or hearing impairment or no friend or family member available to supervise the remote assessment for safety. The study was approved by the local ethics committee, and all participants provided informed consent prior to participation.

An a priori sample size calculation indicated that 36 participants were required, based on an expected intraclass correlation coefficient (ICC) of 0.75, a required precision of 0.15, a 95% confidence level and 5% expected dropout.

### Study procedures

Demographic and health information (age, gender, living arrangements, education, medical history, digital literacy, pain, distress, fatigue and physical activity) was collected through a 15-minute questionnaire. Digital literacy was self-reported on a 5-point scale (‘extremely’ to ‘not at all’) in response to the question ‘How familiar are you with using technology such as a smartphone or computer?’ Participants were randomly assigned (block sizes 2–4), to complete either the remote or in-person assessment first, with the second assessment 1–4 days later (median 2 days). Different assessors conducted each assessment and were blinded to prior scores, reflecting real-world clinical practice. No friends or family attended the in-person assessments.

All remote assessments were conducted using Coviu or Zoom, according to participant preference and were not recorded. Assessors, trained in telehealth-based physical assessments, followed standardised protocols (see Appendix 1) and were familiar with both platforms. Participants received email instructions covering technology, space setup, and safety, and were offered a measuring tape to mark distances for the TUG and walking tests (all already had one). A preparatory call was conducted to test platform access and troubleshoot issues. During the assessment, the device (usually a laptop) was positioned 2–3 metres away to allow a full-body view. Assessors demonstrated each task and gave real-time guidance to maintain visibility and safety. Friends or family members attended to supervise for safety and help with technology; data collection was performed by assessors.

*Feasibility* was evaluated based on the proportion of participants who encountered technological limitations (e.g. lack of a suitable device or internet access) or environmental constraints (e.g. inadequate space). Remote assessments were deemed feasible if fewer than 20% of participants encountered such issues. *Safety* was assessed by recording adverse events occurring during assessments, defined as any untoward medical occurrence (e.g. falls, dizziness, emotional distress), regardless of causality.

### Physical function assessments

Physical function was assessed using five standardised tests. The *five times Sit-to-Stand* (5STS) [11] required participants to rise from a chair five times as quickly and as safely as possible with arms crossed. The time taken to complete the test was recorded. The *Timed Up and Go* (TUG) test [12] involved standing from a chair, walking 3 meters, turning, returning and sitting back down. The time taken to complete the test was recorded. *Standing balance* was evaluated barefooted with eyes open in five progressively challenging positions (feet together, semi-tandem, tandem, left foot, right foot) for 30 seconds each [13]. If participants held a position for 15 seconds or longer with their eyes open, they repeated the test with their eyes closed. The duration of each condition was recorded and summed, with a maximum score of 300 seconds. *Walking speed* was assessed over 4 meters from a standstill on a level surface at their normal walking pace [14]. The time taken to complete the distance was recorded. The Short Physical Performance Battery (SPPB) [15] was used as a composite measure of physical function across standing balance, walking speed over 4 meters and the 5STS, producing a composite score, ranging from 0 to 12, with higher scores indicating better physical function. Remote protocols are provided in Appendix 1 in the Supplementary Data section.

### Data analysis

Data normality was assessed using histograms and Q-Q plots. Non-normal distributions were observed for TUG and SPPB scores, while other measures followed a normal distribution. Within-person differences in scores between settings did follow a normal distribution. To evaluate agreement between remote and in-person assessments, linear mixed-effects models were fitted using the *glmmTMB* function in the statistical software package R (version 2024.12.0 + 467), with location (remote vs in-person) as a fixed effect and participant ID as a random effect. To explore whether recent familiarity with the test affected performance, an additional analysis was run, including order (home vs remote first) and an interaction between location and order as additional fixed effects. Model assumptions were verified using *DHARMa*. The two-way mixed effects ICCs (absolute agreement) were calculated using the *icc* function of the *psych* library. ICCs were interpreted as poor (<0.5), moderate (0.5–0.75), good (0.75–0.90) or excellent (>0.90) [16]. The smallest detectable difference (SDD) was calculated as 1.96 × √2 × SEM to assess test sensitivity across settings. To assess categorical agreement based on clinical cut-offs, the *kappa2* function of the *irr* library was used to calculate Cohen’s kappa for 5STS (cut point ≤15 sec [17]), TUG (cut point ≤10 sec [18]), 4 m Walk (cut point ≤0.8 m/s [6]) and SPPB (cut point ≤8 points [6]). Kappa values were interpreted as poor (≤0.40), moderate (0.41–0.60), substantial (0.61–0.80) or almost perfect (>0.80) agreement [19]. Statistical significance was set at *P* < 0.05.

## Results

Thirty-seven participants (mean age 74.9 years, standard deviation, SD = 6.1; 64.9% women) completed the study (Table 1). They were well-educated and reported being ‘extremely’ to ‘moderately’ familiar with using smartphones and computers, based on a 5-point scale assessing perceived familiarity. Participants had a mean Charlson Comorbidity Index score of 4.0 (SD = 1.9), used an average of 2.9 (SD = 2.4) prescription medications daily and reported a mean EQ-5D-5L VAS score of 86.0 (SD = 9.9). Just over half (56.8%) reported at least one fall in the past year.

**Table 1:** Demographic characteristics of the participants. Values are means (standard deviation) unless stated otherwise

	N = 37
Age, years	74.9 (6.1)
Gender, N (%)	24 (64.9%) women, 13 (35.1%) men
Education, years	18.1 (3.4)
Familiarity with smartphone and computer technology, 1 extremely - 5 not at all	1.7 (0.7)
Charlson Comorbidity Index, 0–24	4.0 (1.9)
Prescription medication, number per day	2.9 (2.4)
EQ-5D-5L VAS Health today, 0–100 best	86.0 (9.9)
Walking aid use, N (%)	1 (3.2%)
Falls in last 12 months, N (%)	21 (56.8%)

### Feasibility

Of the 52 people screened, 37 were eligible and completed both assessments. Exclusions were due to lack of a support person (*n* = 4), inability to travel to the laboratory (*n* = 5), involvement in another study (*n* = 1) and lost interest (*n* = 5). All 52 participants had access to a device and a suitable home environment for remote assessments, including a chair, adequate space and a 4-meter walking area. Chair heights at home ranged from 44 to 50 cm and were measured with a tape measure (self-reported and confirmed visually) during the assessment). Laboratory chairs for the 5STS and TUG were 43 cm (no armrests) and 45 cm (with armrests), respectively. Minor technical issues occurred in three remote assessments (8.1%): two involved loss of audio, resolved by phone call, and one involved video failure, resolved by switching devices. These did not prevent assessment completion.

### Safety

There were no adverse events during any assessment. All participants reported feeling safe during remote procedures and no environmental disruptions were reported.

### Agreement

**Figure 1:**
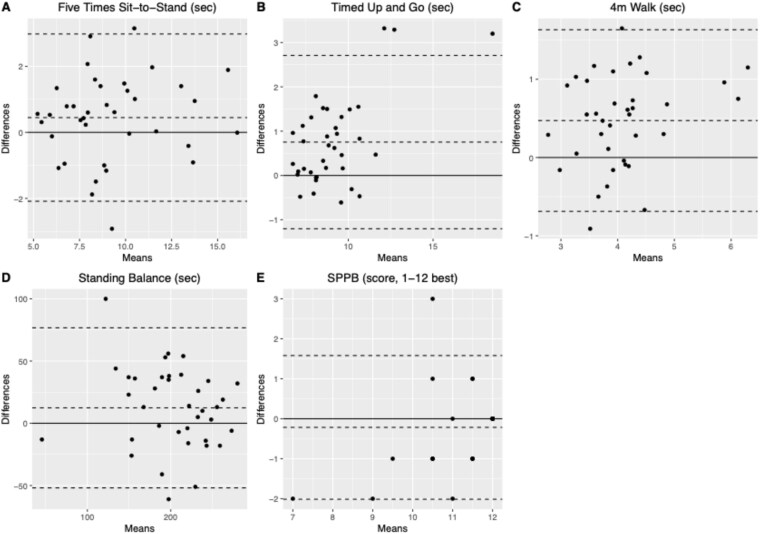
A:E: Bland–Altman plots showing the agreement between in-person laboratory and remote home assessments.

Participants performed better in-person compared to remotely (Table 2, Figure 1), with significant mean differences for the STS (0.5 seconds (95% confidence interval [0.03–0.7], *P* = 0.033), TUG (0.8 [0.4–1.1] seconds, *P* ≤ 0.0001), 4 m Walk (0.5 [0.3–0.7] seconds, *P* ≤ 0.0001), and standing balance (12.4 [2.0–22.8] seconds, *P* = 0.020). SPPB scores did not differ significantly between settings (mean difference: −0.2 [−0.5–0.1] points, *P* = 0.146). Recent familiarity with the test did not significantly affect scores (p_location:order_ = 0.140–0.761), except for standing balance (p_location:order_ = 0.015), where participants who started with the remote assessment showed similar performance between settings (mean difference − 2.1 [−14.9–10.6], *P* = 0.743), while participants who started with the in-person assessment had better performance in-person compared to remote (mean difference: 22.3 [8.6–35.1] seconds, *P* = 0.001).

**Table 2:** Average scores for in-person laboratory and remote home assessments and their agreement

Test	In-person Mean (SD) or Median [IQR]	Remote Mean (SD) or Median [IQR]	Difference Mean [95% CI], *P*	Agreement ICC [95% CI]	Smallest detectable difference
5 Times Sit to Stand (s)	9.0 (2.8)	9.5 (2.9)	0.5 [0.03–0.7], *P* = 0.033	0.89 [0.79–0.94]	1.8
Timed Up and Go (s)	8.4 [2.2]	9.2 [2.2]	0.8 [0.4–1.1], *P* ≤ 0.0001	0.85 [0.54–0.94]	1.4
4 m Walk (s)	3.8 (0.8)	4.3 (0.9)	0.5 [0.3–0.7], *P* ≤ 0.0001	0.64 [0.19–0.84]	0.5
Standing balance (s)	197.0 (53.5)	209.0 (48.2)	12.4 [2.0–22.8], *P* = 0.020	0.77 [0.59–0.88]	32.8
SPPB (score, 1–12 best)	12.0 [1.0]	12.0 [1.0]	−0.2 [−0.5–0.1], *P* = 0.146	0.68 [0.46–0.82]	0.7

SD: standard deviation, IQR: interquartile range, 95% CI: 95% confidence interval, ICC: intraclass correlation coefficient two-way mixed effects absolute agreement, s: seconds, SPPB: Short Physical Performance Battery.

Agreement between remote and in-person scores was good for the 5STS, TUG and standing balance (ICCs 0.89 [0.79–0.94], 0.85 [0.54–0.94], and 0.77 [0.59–0.88], respectively) and moderate for the 4 m Walk and SPPB (ICCs 0.64 [0.19–0.84] and 0.68 [0.46–0.82], respectively). The SDD were 1.8 seconds (5STS), 1.4 seconds (TUG), 0.5 seconds (4 m Walk), 32.8 seconds (standing balance) and 0.7 points (SPPB). Agreement between settings based on clinical cutpoints was substantial for 5STS (κ = 0.65, *P* < 0.001), TUG (κ = 0.77, *P* < 0.001) and SPPB (κ = 0.65, *P* < 0.001), and poor for walking speed (κ = 0.32, *P* = 0.028).

## Discussion

This study evaluated the feasibility, safety, and agreement between in-person laboratory and remote home assessments of physical function in older people. Feasibility was high, with 8% of participants encountering minor technical issues (all resolved) and all screened participants had access to the required space and equipment at home. Importantly, five otherwise eligible participants were excluded due to an inability to attend the laboratory, underscoring the value of remote assessment options. While only a small proportion (7.7%) were excluded due to the lack of a support person, this highlights the need to address safety-related barriers in clinical implementation. Remote assessments proved to be a viable alternative, with no adverse events reported and all participants feeling safe. Agreement between remote and in-person assessments varied across tests, underscoring the importance of careful interpretation in clinical and research settings. Participants generally performed better in the laboratory than at home, possibly due to the more controlled environment and minimal distractions. In addition, unfamiliarity with remote assessments by both participants and assessors may contributed to lower performance at home.

The 5STS and TUG demonstrated good agreement (ICCs = 0.89 and 0.85, respectively), and between-setting differences (0.5 and 0.8 seconds, respectively) below the minimal clinically important differences (MCID) of 1 second or 10% on the 5STS [20] and 2.1 seconds or 1.5 SD on the TUG [21]. These findings align with previous research validating the remote use of the TUG in older people [10] and strengthen the evidence for remote use of the 5STS [22]. In addition to score-level agreement, we evaluated categorical agreement using clinical cut-offs for 5STS and TUG which showed substantial agreement between the settings (κ = 0.65 and 0.77, respectively). Our findings suggest that both tests can be used interchangeably across remote and in-person settings for clinical decision-making and monitoring progress.

In contrast, standing balance showed a good agreement (ICC = 0.77) but the SDD between settings was 32.8 seconds, which is relatively large in relation to typical intervention effects. While the between-setting difference of 12.4 seconds exceeded the 10-second improvement observed in a 12-month balance exercise intervention [8], the SDD suggests this test may be less sensitive to detecting smaller, clinically relevant changes across settings. Moreover, the effect of the order of the assessments, where participants who first completed the in-person assessment performed better in that setting than those who started remotely, suggests a potential role for priming. Experiencing the assessment first in a controlled laboratory setting may have set expectations, confidence or task interpretation in ways that did not carry over to the remote setting and deserves further exploration in a larger sample. Protocol refinements or use of advanced technology to measure, e.g. sway could improve its sensitivity in remote contexts.

Walking speed via the 4 m Walk test showed moderate agreement (ICC = 0.64 and κ = 0.32) and the between-setting SDD (0.5 seconds) exceeded the MCID of 0.12–0.2 s over 4 metres [23]. This variability highlights that self-selected walking speed is highly context-dependent, influence by instruction and setup, and sensitive to small timing or distance errors in home settings [24, 25]. Future research should explore alternative instructions (e.g. walking at your fastest speed) or use technological solutions, such as computer vision or wearable sensors [26], to improve the reliability of remote walking speed assessments.

SPPB also showed moderate agreement between settings (ICC = 0.68), largely driven by variability in walking speed, while standing balance and 5STS components alone showed good agreement. Although the average between-setting difference (−0.2 points) was below the MCID (0.3–0.8 points) [23, 27] and clinical agreement was substantial (κ = 0.65), caution is warranted when interpreting SPPB scores remotely, as variations in walking speed may affect total scores and complicate their use for monitoring or comparing functional changes across settings.

This study has some limitations. First, recruitment via an online volunteer database likely selected older people with moderate to high digital literacy, limiting generalisability to those less familiar with technology. Second, while home environments were confirmed as suitable, flooring type was not formally recorded, and chair height varied. Third, the study was conducted at a single site and may not fully capture the diversity of home environments, cultural contexts or healthcare systems. Finally, as each test was conducted once per setting, responsiveness to change was not directly assessed and reported SDDs reflect only measurement reproducibility rather than longitudinal sensitivity.

These findings have important clinical and research implications for implementing telehealth into routine practice for assessing physical function in older people. Telehealth offers accessible and cost-effective alternatives to in-person assessments, especially for older people with limited mobility, transportation barriers, or those living in regional or remote areas. The need for remote assessment options is underscored by the number of otherwise eligible participants in this study who were unable to attend the clinic. While remote assessments showed good overall agreement, clinicians should interpret measures like walking speed with caution, particularly when small changes may influence clinical decisions. The demonstrated feasibility and safety support broader implementation of telehealth into clinical practice and research, provided that protocols are refined and barriers, such as the need for a support person, are addressed to ensure equitable access. While this study focused on replicating traditional clinical parameters (e.g. duration, scores), smartphones and sensor-based tools offer new possibilities to enhance both remote and in-person assessments. These include real-time monitoring of postural sway, gait variability and mobility patterns, with potential to deliver more sensitive and ecologically valid measures of physical function [26]. Given that smartphones are widely owned and carried, they offer a scalable platform for continuous, less assessor-dependent assessment. Realising this potential will require the development and clinical validation of standardised algorithms [28].

In conclusion, remote assessments of physical function in older people was found to be feasible and safe, with no adverse events reported and high completion rates despite minimal technical issues. Good agreement was observed between remote and in-person assessments for the 5STS, TUG and standing balance, supporting their potential use in routine telehealth settings. Agreement was lower for walking speed and the SPPB. While these tests may still be informative when used remotely, caution is needed when interpreting small changes or classifying functional risk based on threshold scores. These findings support the use of remote assessments for monitoring lower-limb strength, balance and mobility in older people, especially when in-person testing is not possible. As telehealth becomes more embedded in practice, future research should refine remote protocols and explore technology-assisted solutions, such as smartphone-based assessments, to improve measurement precision and support broader implementation.

## Supplementary Material

aa-25-0592-File002_afaf266

## Research Data Transparency and Availability

Data are available from k.vanschooten@neura.edu.au upon reasonable request.
