# Supplementary material for: Remote assessment of physical function in older people: feasibility, safety and agreement with in-person administration
Source: Age Ageing. 2025 Sep 27;54(9):afaf266. doi: 10.1093/ageing/afaf266 (PMC12476136; doi:10.1093/ageing/afaf266)
Supplement: aa-25-0592-File002_afaf266 [file aa-25-0592-file002_afaf266.docx]

**Appendix 1: Remote Physical Function Assessment Protocol via Teleconference**

**Pre-Assessment Preparation**

**Assessor**: Ensure the following before the assessment begins:

- - Personal phone on silent and set to private/no caller ID if needed to call participant.
  - Computer notifications are turned off.
  - Remove glasses if not required for computer viewing.
  - Intermittent eye contact is made by occasionally looking at the camera.

**Participant**: Ensure the following before the assessment begins:

- - Device for video calls (laptop/computer/iPad/tablet).
  - A working camera and microphone on your device.
  - A stable internet connection.
  - Enough space in front of your device to stand and move freely. We will need to be able to see you from head to toe.
  - A support person available to assist with technology or safety, if needed.
  - Wear comfortable shoes and clothing. Pants or shorts are appropriate. Please avoid skirts, dresses, and stockings. Wear secure footwear (no slippers or sandals).
  - Available equipment as detailed below.

**Required Equipment**

- Standard chair (with or without armrests).
- Stopwatch or timing device.
- Measuring tape of at least 4 meters in length. Assessor: Confirm before the assessment whether one needs to be provided.
- Two visible markers (e.g., shoes, sticky notes, water bottles).

**Setting Up the Environment and Camera**

- Ensure the floor is flat and free of obstacles.
- Choose a quiet, well-lit space with minimal distractions.
- Minimise background noise (close windows/doors, notify others in the household).
- Place a chair against a stable background to prevent movement during tests.
- Camera should be set up:
  - On a stable surface (e.g., table, shelf) or mounted on a tripod.
  - At a height that captures the full body for sit-to-stand and balance tests.
  - Wide enough angle to view walking start and end points (adjust or reposition if needed).
- Ensure sufficient space for:
  - 3 metres of straight walking for TUG.
  - 4 metres of walking space for gait speed test.
  - Standing balance positions with access to a chair for safety.

**Assessments**

**1. Five Times Sit-to-Stand Test (5STS)**

**Purpose:** To assess lower limb strength.

**Instructions:**

- Use a standard chair. Participant sits on the edge, feet flat on the floor.
- Arms crossed over chest. If not possible, hands on thighs (document modification).
- Participant performs five full sit-to-stand movements as quickly and safely as possible.
- The assessor or support person times the test.
- Count each repetition aloud.
- Stop timing at the end of the 5th stand.

**Script:** “This is a sit-to-stand test to assess your leg strength. Please sit up straight with your feet flat on the floor. Cross your arms over your chest if you can. If you need to, you may use your hands to push off your thighs or the chair. When I say go, stand up and sit down five times as quickly and safely as you can. Make sure to fully stand and sit each time. Ready, set, go.”

**Telehealth Notes:**

- Please note the inclusion of use of the arms and the instruction on safely here, to manage fall risk.
- Support person adjusts camera angle.
- Support person positioned to the side to avoid blocking the view.
- Test may be demonstrated by assessor if needed.
- Ask participant or support person to measure chair height.

**2. Timed Up and Go (3m-TUG)**

**Purpose:** To assess mobility and fall risk.

**Instructions:**

- Use a standard chair.
- Measure 3 metres from the front of the chair to a marker on the floor.
- Participant wears shoes and uses usual assistive device.
- Arms crossed during the initial stand only, then released.
- Timing starts on “Go” and ends when participant is seated again.

**Script:** “This test assesses your mobility. Start seated with you back against the chair. When I say ‘go,’ stand up from the chair, walk to the marker on the floor at your normal comfortable pace, turn around, walk back to the chair at the same pace, and sit down again with your back against the chair. Ready, set, go.”

**Telehealth Notes:**

- Support person to check 3m distance.
- Ensure clear visibility for camera view at both start and return.
- Repeat if performed incorrectly.

**3. Standing Balance Assessment**

**Purpose:** To assess standing balance.

**Instructions:**

- Performed barefoot.
- Use nearby chair for safety.
- Arms by side, no talking during the test.
- Positions tested in order:
  - Feet together
  - Near tandem (small step forward with one foot)
  - Full tandem (heel-to-toe)
  - One-leg stance (each leg)
- Each stance held for up to 30 seconds.
- Repeat stances with eyes closed only if held ≥15 seconds with eyes open.

**Script:** “This test assesses your standing balance. I’ll ask you to hold different positions for up to 30 seconds without holding on. Please stand near a chair or support. Use the chair to get into position and let go when ready. I’ll time once you let go. If you feel unsafe, hold on or return to a stable position.”

**Eyes Closed Variation:** “Because you held some positions for more than 15 seconds, we’ll now repeat those positions with your eyes closed. Close your eyes only once you feel stable.”

**Foot Position Definitions:**

- **Feet Together:** Feet touching or as close as possible; knees may touch first.
- **Near Tandem:** Small diagonal step forward; ~2cm gap between feet.
- **Full Tandem:** Heel-to-toe; like a tightrope walker.
- **Single Leg:** Lift one foot just off the floor; do not rest against standing leg.

**Telehealth Notes:**

- Support person to check foot position.
- Use hand gestures to demonstrate foot positions.
- Chair placement and participant stability must be visually confirmed.

**4. 4-Metre Walk Test (4MWT)**

**Purpose:** To assess gait speed.

**Instructions:**

- Measure and mark a 4m walkway.
- Participant starts at 0m and walks to 4m marker.
- Usual walking pace.
- Performed twice.

**Script:** “This is a walking test. Stand at the starting marker and walk to the end marker at your normal pace when I say ‘go.’ This is not a race. Just walk as you usually do. We’ll do this twice. Are you ready? Set, go.”

**Telehealth Notes:**

- Please note the walking stopped at 4m because of space constraints at home.
- Support person to adjust camera angle.
- Only end of walkway needs to be visible.
- Use visible household items as markers.

**Additional Considerations**

- Support person may be asked to time tests or assist with safety and camera adjustments.
- Assessor and support person coordinate closely to minimise issues due to video lag.
- Record any deviations from standard procedure (e.g., use of arms in 5STS, not barefoot in balance test).
- Assessors should prompt participants clearly and calmly, using visual cues when needed.
